# Unexpected Coronary Sinus Embolization of a Leadless Pacemaker due to Initial Device Failure: A Case Report

**DOI:** 10.1002/ccr3.70984

**Published:** 2025-09-29

**Authors:** Yutaro Oshima, Tsuyoshi Nozue, Masahiro Katamine, Taku Iwaki

**Affiliations:** ^1^ Department of Cardiology Yokohama Sakae Kyosai Hospital Yokohama Kanagawa Japan

**Keywords:** coronary sinus, device failure, device retrieval, embolization, leadless pacemaker

## Abstract

Leadless pacemakers reduce complications compared to transvenous systems, but device dislodgement and embolization can occur. We report an 81‐year‐old man with atrial fibrillation and bradycardia who underwent Micra implantation. Because of inner shaft damage and insufficient tine fixation, the device embolized into the coronary sinus. It was successfully retrieved using a double‐snare technique, and a new device was implanted. This case highlights the importance of meticulous deployment technique and thorough intraprocedural assessment of device attachment, as well as troubleshooting strategies for unexpected embolization.

## Graphical abstract

A leadless pacemaker embolized into the coronary sinus due to initial device failure. The device was successfully retrieved using a double‐snare technique under fluoroscopic guidance.

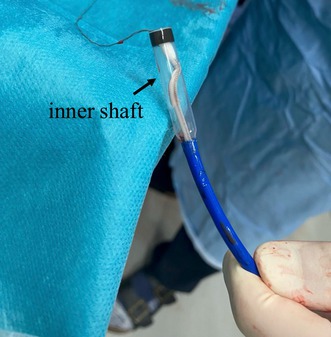

**Summary**

- Leadless pacemaker embolization into the coronary sinus due to device failure is extremely rare.
- This case underscores the importance of meticulous deployment technique and early post‐procedural imaging to detect and manage malposition.

## 1 Introduction

The Micra transcatheter pacing system (TPS) has become as popular as conventional pacemakers. Fewer severe and long‐term complications occur compared with conventional transvenous pacemakers because lead and device pocket creation are not required [1, 2]. However, acute device dropout has been reported, and there is a need to become familiar with their handling. Micra pacemakers have been reported to drop out because of abnormal cardiac morphology or device crimping failure [3, 4]. Herein, we report a case of device dislodgment related to an initial mechanical device failure and successful retrieval.

## 2 Case History/Examination

An 81‐year‐old man was admitted to our hospital with chronic heart failure due to bradycardia and long‐standing persistent atrial fibrillation. The patient presented with dyspnea. Physical examination on admission revealed mild edema of the lower extremities. Transthoracic echocardiography revealed preserved left ventricular systolic function with an EF of approximately 65% and moderate tricuspid regurgitation. His heart rate was persistently low at around 20–30 beats per minute. The patient was diagnosed with worsening heart failure, long‐standing persistent atrial fibrillation, and slow ventricular response. Given the marked bradycardia with long‐standing persistent atrial fibrillation, a leadless pacemaker was indicated [5]. The leadless pacemaker was implanted the day after admission.

**FIGURE 1:**
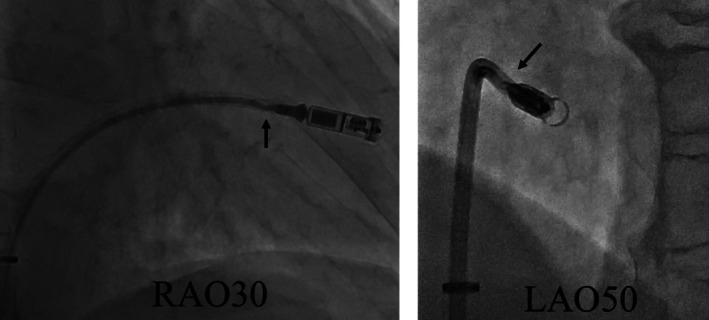
Right anterior oblique 30° and left anterior oblique 30° views demonstrating flexion of the inner shaft prior to deployment.

**FIGURE 2:**
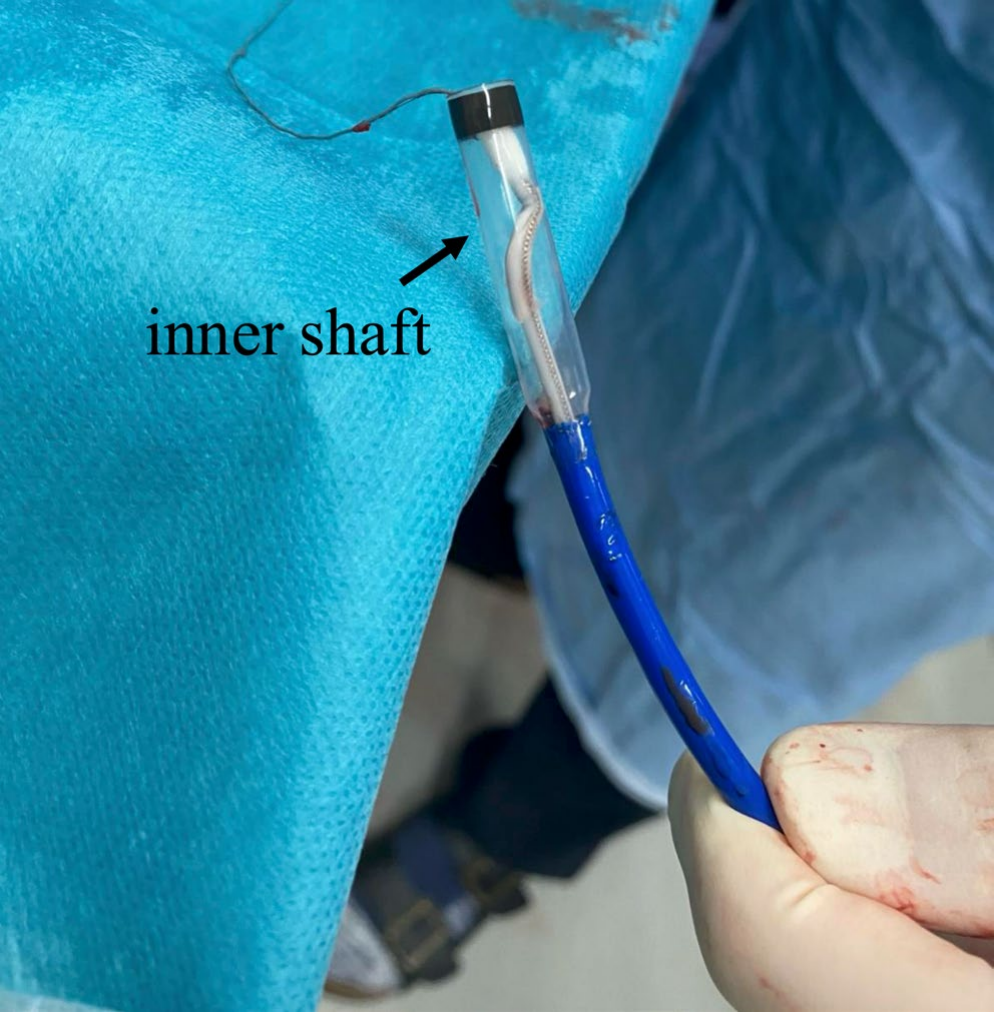
Retrieved transcatheter pacing system (TPS) showing visible shaft damage.

**FIGURE 3:**
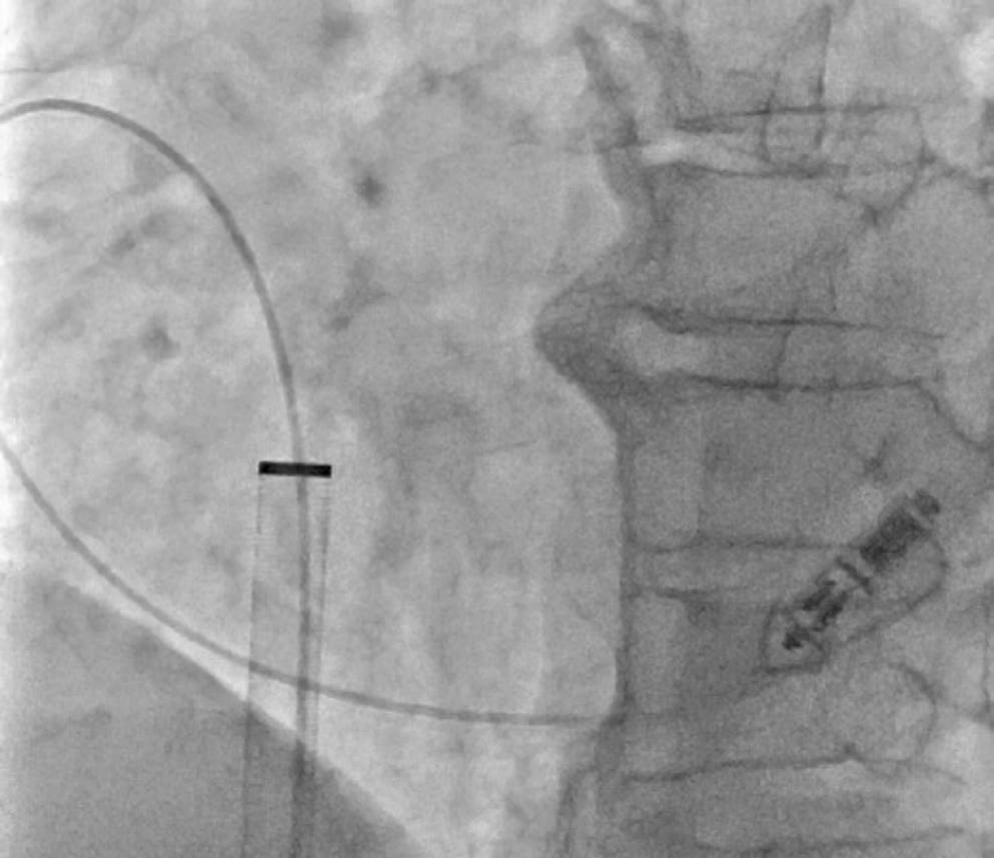
A pigtail catheter advanced into the coronary sinus via an alpha curve to mobilize the embolized device.

**FIGURE 4:**
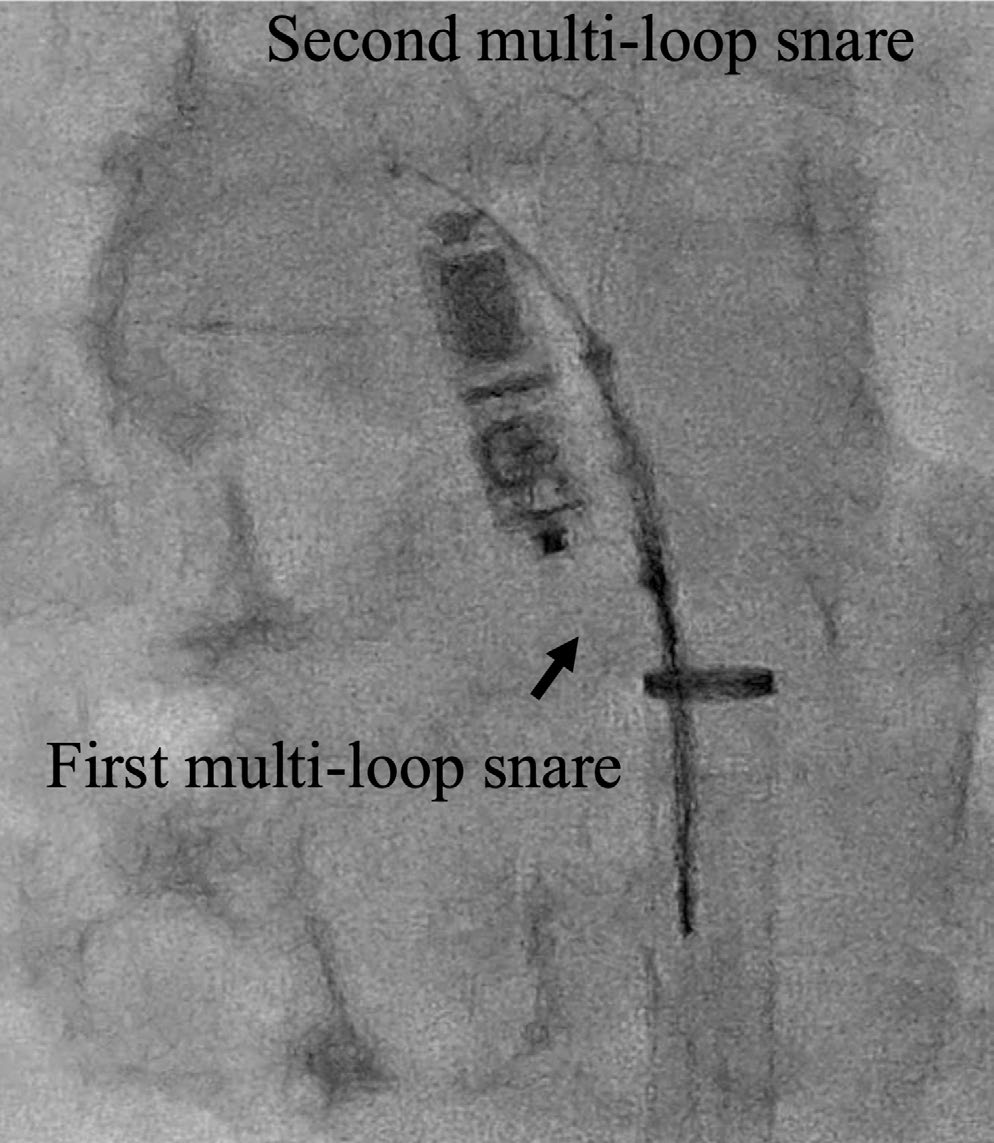
Double‐snare coaxial retrieval. The first snare held the tine, while the second engaged the retrieval feature.

The 23 Fr Micra TPS sheath was inserted into the inferior vena cava (IVC) via the right groin. Although part of the inner shaft appeared to be flexed during implantation, we continued the procedure because we considered it would not affect the procedure (Figure 1). Electrical parameters were as follows: sensing amplitude 6.0 mV, pacing threshold 0.75 V at 0.4 ms, and impedance 560 Ω, confirming adequate function. The tines were believed to be engaged based on pull‐and‐hold testing, though retrospectively, engagement may have been incomplete. After cutting the tether, we attempted to remove it, but it could not be pulled out. Owing to the breakage within the inner shaft, we subsequently attempted to remove the tether; however, complete extraction was considered impossible because the tether had become lodged within the damaged shaft. The tether could not be removed using standard techniques. We then applied considerable traction, which inadvertently caused the Micra pacemaker to surge out of the right ventricle and lodge in the coronary sinus. Once the Micra TPS was retrieved from the body, we noted that the inner shaft was damaged (Figure 2). To reposition the device into the right atrium, we advanced a pigtail catheter with a 0.035″ guidewire into the coronary sinus via an alpha curve, allowing us to hook and mobilize the Micra pacemaker (Figure 3). The Micra pacemaker was successfully removed from the coronary sinus by hooking the tip of the pigtail catheter onto the tine (Video 1). We inserted a steerable introducer (Agilis, Abbott, Abbott Park, Illinois, USA) into the leadless pacemaker sheath and successfully snared the tine with an 18–30 mm multi‐loop snare (EN Snare; Merit Medical Systems, South Jordan, USA). However, it was impossible to retrieve the device into the TPS sheath because of interference from the edge of the leadless pacemaker. We then used the double‐snare technique to achieve coaxial alignment. We stopped the movement of the Micra pacemaker by snaring the tine and snaring the retrieval feature with another 12–20 mm multi‐loop snare, and the device was successfully removed into the sheath (Figure 4 and Video 2). We attempted to re‐implant the new Micra device and successfully placed it in the septal position. The total procedure time was 195 min. Transthoracic echocardiography revealed no pericardial effusion, and the patient was discharged on postoperative day 4. A timeline of the clinical course is summarized in Table 1.

**VIDEO 1:** Pigtail catheter advanced with a 0.035‐in. guidewire into the coronary sinus, successfully mobilizing and extracting the embolized Micra leadless pacemaker. Still frame shown at 7 s. Video content can be viewed at https://onlinelibrary.wiley.com/doi/10.1002/ccr3.70984.

**VIDEO 2:** Double‐snare retrieval of the embolized Micra pacemaker using a steerable sheath. One snare captured a tine, while the second snare engaged the retrieval feature, achieving coaxial alignment and safe withdrawal into the sheath. Still frame shown at 42 s. Video content can be viewed at https://onlinelibrary.wiley.com/doi/10.1002/ccr3.70984.

**TABLE 1:** Timeline of clinical course and procedures.

Day/timepoint	Event
Admission (Day 0)	An 81‐year‐old man was admitted with worsening heart failure, bradycardia, and long‐standing persistent atrial fibrillation
Day 1	A Micra leadless pacemaker was implanted in the right ventricular septum
During procedure	Inner shaft damage caused tether removal failure. Excessive traction resulted in device embolization into the coronary sinus.
Same procedure	Device mobilized from the coronary sinus using a pigtail catheter, then successfully retrieved with a double‐snare technique
Same procedure	A new Micra device was re‐implanted in the septal position with stable parameters
Day 4	Patient discharged without complications
Follow‐up	Device parameters remained stable and the patient was asymptomatic

## 3 Differential Diagnosis, Investigations and Management

### 3.1 Differential Diagnosis

Device dislodgment secondary to tether failure, right ventricular anatomical instability such as trabecular irregularity or atypical morphology preventing stable tine engagement, or improper tine fixation.

### 3.2 Investigations

Fluoroscopy and intraprocedural imaging confirmed embolization into the coronary sinus.

### 3.3 Management

1. A 0.035″ guidewire‐supported pigtail catheter was advanced via an alpha curve into the coronary sinus to mobilize the device (Figure 3 and Video 1).
2. An initial single‐snare attempt failed.
3. A steerable introducer (Agilis, Abbott, USA) and two multi‐loop snares were then used in a double‐snare coaxial technique—one snared the tine and the other snared the proximal retrieval feature—to stabilize and recapture the device into the sheath (Figure 4 and Video 2).
4. A new Micra device was subsequently redeployed in the septal position.
5. The total procedure time was 195 min, and postoperative echocardiography confirmed no pericardial effusion. The patient was discharged on Day 4.

## 4 Results (Outcome and Follow‐Up)

Percutaneous retrieval of the embolized device was successful without complications. The re‐implanted Micra functioned normally. The patient remained asymptomatic, with normal device parameters and no evidence of pericardial effusion. He was discharged on postoperative day 4. Further follow‐up showed stable clinical status.

## 5 Discussion

To the best of our knowledge, we report a rare case characterized by initial mechanical device failure and the unusual occurrence of a leadless pacemaker becoming lodged in the coronary sinus.

Dislodgement can be caused by anatomical abnormalities of the right ventricle, such as cardiac amyloidosis and unstable “cliffhanger” condition [3, 4, 6]. In our case, the tether could not be extracted owing to the initial breakage of the inner shaft. The most important finding of this case is that initial mechanical device failure is sporadic; however, it is associated with a critical complication. The defective catheter was sent back to Medtronic for further analysis, but we have not yet received an official response regarding the cause of the failure, and the device is still being analyzed by Medtronic, with results pending.

Another unique finding of this case was that the device was embolized into the coronary sinus. Although there have been several case reports of pacemakers becoming stuck in the pulmonary artery [3, 7, 8, 9, 10], cases of pacemaker embolization into the coronary sinus are extremely rare, and to the best of our knowledge, only one other case has been reported besides ours [10]. In this case, favorable device orientation facilitated retrieval, whereas opposite orientation could have necessitated surgery; therefore, preventive strategies such as meticulous tine engagement and stability confirmation before tether release are essential. It was possible to advance the pigtail catheter into the coronary sinus; however, this may not always be possible. The cardiac resynchronization therapy delivery system would make it easier to reach the device (guidewire or multi‐loop snare) into the coronary sinus. However, it requires an additional puncture site in the subclavian or jugular vein.

The double‐snaring technique was effective in achieving coaxial alignment between the device and the sheath. There are two approaches to the double‐snaring technique: two‐puncture two‐directional approach [6] and one‐puncture two‐directional approach.

Hasegawa‐Tamba et al. reported that a two‐puncture two‐directional approach with the superior vena cava (SVC) and IVC is easier to operate in some situations because they do not interfere with each other and may have advantages [6]. However, the two‐puncture two‐directional approach requires an additional puncture site and increases the risk of hemorrhagic complications. Therefore, the one‐puncture two‐directional approach is a useful option when the two snares do not interfere with each other, thereby avoiding the risk of bleeding complications.

## 6 Conclusion

A leadless pacemaker system is a very useful tool; however, it rarely causes initial mechanical device failure. Careful attention should be paid to initial mechanical device failure because it may be associated with severe complications.

## Author Contributions

**Yutaro Oshima:** conceptualization, data curation, investigation, supervision, visualization, writing – original draft, writing – review and editing. **Tsuyoshi Nozue:** investigation. **Masahiro Katamine:** investigation. **Taku Iwaki:** investigation.

## Disclosure

The authors have nothing to report.

## Consent

Written informed consent was obtained from the patient to publish this report in accordance with the journal's patient consent policy.

## Conflicts of Interest

The authors declare no conflicts of interest.

## Data Availability

The authors have nothing to report.
